# Ultrasonographic scores for ileal Crohn’s disease assessment: Better, worse or the same as contrast-enhanced ultrasound?

**DOI:** 10.1186/s12876-022-02326-6

**Published:** 2022-05-18

**Authors:** M. Freitas, F. Dias de Castro, V. Macedo Silva, C. Arieira, T. Cúrdia Gonçalves, S. Leite, M. J. Moreira, J. Cotter

**Affiliations:** 1 Gastroenterology Department, Hospital da Senhora da Oliveira, Guimarães, Portugal; 2 Life and Health Sciences Research Institute (ICVS), School of Medicine, University of Minho, Braga, Portugal; 3 ICVS/3B’s, PT Government Associate Laboratory, Braga/Guimarães, Portugal

**Keywords:** Ultrasound, Contrast-enhanced ultrasound, Crohn’s disease, Inflammatory bowel disease

## Abstract

**Background:**

Intestinal ultrasound (IUS) is an increasingly used non-invasive tool to evaluate Crohn’s disease (CD) activity. Recently, two IUS scores that evaluate inflammatory activity have emerged: the Simple Ultrasound Activity Score for CD (SUS-CD) and the International Bowel Ultrasound Segmental Activity Score (IBUS-SAS). We aimed to compare the accuracy of SUS-CD, IBUS-SAS and contrast-enhanced ultrasound (CEUS) in predicting inflammatory activity in the terminal ileum in ileocolonoscopy in CD patients.

**Methods:**

Retrospective study including all consecutive CD patients submitted to IUS with CEUS directed to the terminal ileum performed by a single operator between April 2016 and March 2020. Segmental SUS-CD and IBUS-SAS were calculated. A time-intensity curve of the contrast bowel wall enhancement was created with measurement of peak intensity using CEUS. The CD endoscopic activity in ileocolonoscopy was graded by Simple Endoscopic Score for CD (SES-CD) as inactive (SES-CD < 7) or active (SES-CD ≥ 7).

**Results:**

Fifty patients were included, 54.0% were female, with mean age of 34 ± 12 years, and most had isolated ileal disease (60.0%), and a nonstricturing, nonpenetrating behaviour (44.0%). Most of the patients (60.0%) had active endoscopic disease (SES-CD ≥ 7). SUS-CD and IBUS-SAS were not different between patients with active or inactive endoscopic disease (*p* = 0.15; 0.57, respectively), having a poor accuracy to correlate endoscopic activity (area under de curve (AUC) 0.62; 0.55, respectively). Peak intensity in CEUS was significantly different in patients with active or inactive endoscopic disease (*p* = 0.004), having a good accuracy to correlate endoscopic activity (AUC 0.80).

**Conclusion:**

Unlike CEUS, SUS-CD and IBUS-SAS were not able to accurately correlate endoscopic activity in terminal ileum in CD. Therefore, CEUS is a non-invasive emerging method that should be increasingly integrated in the ultrasonographic evaluation of CD patients.

## Introduction

Intestinal ultrasound (IUS) has become one of the most valuable progresses of ultrasound in the past decade, especially for patients with inflammatory bowel disease (IBD), that due to its chronic fluctuating course, require frequent monitoring of inflammatory activity [1–3].

Nowadays the standard of care in Crohn’s disease (CD) is guiding management based on objective evaluations of disease activity instead of patient’s symptoms, as recommended by current guidelines [2–4], since clinical assessment correlates poorly with mucosal inflammation [5–8] and long-term outcomes improve by establishing therapeutic goals based on objective parameters [9].

Ileocolonoscopy is the gold standard method for diagnosis and monitoring of CD activity, although it cannot be performed on a regular basis since it is invasive, with some known complications, resource intensive, uncomfortable for the patient and it is not always technically possible to perform cecal and/or ileal intubation [2, 3].

Cross-sectional imaging, such as computed tomography (CT) and magnetic resonance imaging (MRI), and IUS are non-invasive, and are increasingly recognized as important diagnostic and monitoring tools in CD management [10]. The ECCO-ESGAR guidelines recommends both magnetic resonance enterography (MRE), IUS and capsule endoscopy as first-line modalities for small bowel assessment in CD patients, given their accuracy and absence of ionizing radiation exposure [3]. A study comparing IUS and MRE performed in 234 consecutive patients with suspected CD showed a similar diagnostic accuracy in detecting small bowel CD [11]. MRE use can be limited by low accessibility, time consuming acquisition times, in some cases poor patient acceptance, necessity of specific preparation and high costs [12].

Inflammation biomarkers such as C-reactive protein (CRP) and faecal calprotectin are used as a complement of the other additional examinations in the diagnosis and disease monitoring [2, 3], since they cannot evaluate disease location and extension and have limited accuracy and specificity [13].

IUS has high accuracy to assess CD activity [14] and to monitor disease activity in response to medical treatment [15], and has the advantages of being non-invasive, having immediate availability within the clinical setting as it can be performed bedside, being a well accepted and tolerated examination, with an easy repeatability, absence of ionizing radiation, sedation or preparation, and having a low cost [3, 16, 17]. Besides, patient preference is an important issue to consider [18, 19]. However, conventional and doppler IUS are highly operator dependent, and require training and expertise [14].

Although, currently IUS is an increasingly used tool to monitor CD activity, there is no widely accepted reproducible IUS activity index to evaluate inflammatory activity, since the methodology for development of the scores was shown to be insufficient in most studies and none have been adequately validated [20]. In 2021, two new scores were published: the Simple Ultrasound Activity Score for CD (SUS-CD) [21] and International Bowel Ultrasound Segmental Activity Score (IBUS-SAS) [22]. Both include evaluation of the terminal ileum, colon and rectum. SUS-CD is a validated score that includes the IUS evaluation of bowel wall thickness (BWT) and colour Doppler signal (CDS), and showed a good correlation with endoscopic disease activity [21] IBUS-SAS includes evaluation of BWT, bowel wall stratification (BWS), CDS and inflammatory mesenteric fat (i-fat) and predicted endoscopic disease activity [22].

Contrast-enhanced ultrasound (CEUS) is an emerging method that involves microvessel passage of an intravenously administrated microbubble contrast agent, providing information on local tissue vascularization and perfusion. Increased bowel wall perfusion indicates active inflammation [23, 24]. CEUS has shown a good sensitivity (94.0%) and moderate specificity (79.0%) in the detection of active CD [25]. It is important to understand if CEUS is superior to conventional IUS in assessing disease activity, in order to optimize monitoring of CD patients.

Our aim was to compare the accuracy of SUS-CD score, IBUS-SAS score and CEUS in predicting inflammatory activity in the terminal ileum in ileocolonoscopy in patients with CD.

## Materials and methods

### Patients and data collection

We performed a retrospective single-center study including all consecutive CD patients submitted to conventional IUS and CEUS directed to the terminal ileum in the Gastroenterology Department of an University affiliated Hospital between April 2016 and March 2020.

Demographic, clinical, biochemical, endoscopic and ultrasonographic data were collected by reviewing medical records and included patients’ age and gender, age at diagnosis, location and behaviour of CD according to Montreal classification [26], C-reactive protein (CRP) and fecal calprotectin, and ileocolonoscopy and ultrasonographic findings. The Harvey Bradshaw Index (HBI) [27] was calculated to assess clinical disease activity, and clinical remission was defined as HBI < 5, as previously recommended [28]. Elevated fecal calprotectin was defined as ≥ 150 μg/g, as previously suggested [29], and elevated CRP was defined as > 3.0 mg/L, based on the reference value of our institution.

Only biochemical and ileocolonoscopy data performed with a maximum interval of 1 month until the ultrasonographic examinations were considered.

Ileocolonoscopy and ultrasound examinations were performed as a part of regular follow-up, including suspicion of active disease, assessment of remission or relapse, and monitoring of treatment effect.

Paediatric age, pregnancy, previous surgery evolving terminal ileum, CD without ileal involvement (L2 of Montreal classification [26]) were considered exclusion criteria. Additionally, patients with inadequate bowel preparation during ileocolonoscopy, or in whom ileon intubation was not possible, or with inadequate ultrasonographic profile that did not allow adequate observation of the terminal ileum, were not included in the study.

### Ileocolonoscopy

Endoscopic examinations were performed by an experienced endoscopist with expertise in the diagnosis, surveillance, activity assessment and endoscopic treatment of IBD and its complications. The CD activity was assessed with ileocolonoscopy (reference) by segmental Simple Endoscopic Score for CD (SES-CD) [30] applied to the terminal ileum. The SES-CD evaluates four endoscopic variables: ulcer size, ulcerated surface, affected surface and stenosis. Each parameter has a value of 0–3 according to its severity, and by summing the points, segmental endoscopic activity of terminal ileum was quantified.

The disease activity was graded as inactive (normal or mild disease, with a SES-CD < 7) or active (moderate or severe disease, with a SES-CD ≥ 7), as previously defined in several studies [31–34].

### Ultrasound examination

Ultrasonographic examinations were performed by a single expert operator experienced in IUS (> 100 examinations) that is faculty member and instructor in IUS-specific courses, using an ultrasound Hitachi HI VISION Avius® UST-9130 (Hitachi Medical Corporation, Tokyo, Japan), employing a convex, low frequency (1–5 MHz) and linear, high frequency (5–13 MHz) transducers directed to the terminal ileum.

The ultrasound operator did not perform ileocolonoscopy examinations and the ileocolonocopy examinations were performed after ultrasonographic examinations by an operator without experience in ultrasound. There were no changes in medical therapy between the examinations.

Patients were submitted in the same examination, first to conventional IUS (B-mode and Doppler) and then to CEUS with contrast SonoVue® (Bracco UK). Qualitative and quantitative parameters from the conventional IUS analysis including BWT, BWS, CDS and i-fat were evaluated.

BWT measurements were performed in longitudinal and cross-section orientations and two measurements in each orientation were obtained. For the SUS-CD score the average of two measurements in longitudinal orientation was considered and for the IBUS-SAS score the average of 4 measurements (two in longitudinal and two in cross-section orientation) was considered. Loss of bowel wall stratification was defined as a hypoechoic disruption of the 3 distinct wall layers that characterize a normal bowel wall stratification. Colour Doppler acquisitions were performed using standardized scanning pre-sets during patient breath-hold. The velocity scale was set to 5 cm/s, enabling registration of vessels with low velocities. Gain was turned up to a level where flash artefacts occurred, and then lowered until they disappeared. Evaluation of colour Doppler was performed for SUS-CD assessment using a modified version of that of Spalinger et al. [35] counting the number of Doppler signals per cm^2^ and for IBUS-SAS score assessment using a modified Limberg score, assessing the detectable colour Doppler signals/pixels inside and outside the bowel wall (Table 1). i-fat was defined as a homogeneous, hyperechoic changes around thickened bowel wall.

**Table 1:** IUS scores and CEUS parameters

SUS-CD [21]	BWT + CDS			
	0	1	2	3
BWT	< 3.0 mm	3.0–4.9 mm	5.0–7.9 mm	≥ 8.0 mm
CDS	No or single vessel	2–5 vessels/cm^2^	> 5 vessels/cm^2^	-

IBUS-SAS [22]	4 × BWT + 15 × i-fat + 7 × CDS + 4 × BWS			
BWT	Quantitative measure in mm			
	0	1	2	3
i-fat	Absent	Uncertain	Present	-
CDS	Absent	Short signals	Long signals inside bowel	Long signals inside and outside bowel
BWS	Normal	Uncertain	Focal (≤ 3 cm)	Extensive (> 3 cm)

CEUS	Peak intensity			
Peak intensity	Quantitative measure based on time intensity curve			

*IUS* Intestinal ultrasound; *CEUS* Contrast ultrasound; *SUS-CD* Simple Ultrasound Activity Score for CD; *BWT* Bowel wall thickness; *CDS* Color Doppler signal; *IBUS-SAS* International Bowel Ultrasound Segmental Activity Score; *i-fat* Inflammatory fat; *BWS* Bowel wall stratification.

Segmental SUS-CD [21] and IBUS-SAS [22] scores applied to terminal ileum were calculated using the ultrasound variables previously described, according to the authors (Table 1).

The peak intensity, a quantitative measurement of contrast enhancement of bowel wall, which reflects the bowel wall microvascularity, was evaluated by placing a region of interest (ROI) within the enhanced bowel wall in terminal ileum and by performing time-intensity curve (TIC) analysis using CEUS with contrast SonoVue® (Bracco UK) (Table 1). The contrast constituted by sulphur hexafluoride microbubbles, was injected intravenously in a 2.4 ml dose, immediately followed by injection of 10 mL of normal saline solution flush, as previously recommended [24]. The TICs were generated in the Motion-Compensated Microbubble Trace Imaging (MC-MTI) accumulative enhancement mode, were recorded and analysed using built in software (EZU-CH8) on the ultrasound machine.

### Statistical analysis

Statistical analysis was carried out with SPSS® software version 24.0 (IBM, Armonk, New York, USA). Categorical variables are presented as frequencies and percentages, and continuous variables as mean and standard deviation (SD). Categorical variables were compared using χ^2^-test or Fisher’s exact test (two-tailed) as appropriate. Continuous variables were compared using Student’s t-test. IUS scores and CEUS performance in predicting CD inflammatory activity was evaluated by assessing its discrimination with area under the receiver operating characteristic curves (AUCs), with 95% confidence intervals (CIs). A *p* value of less than 0.05 was defined as statistically significant.

### Ethical considerations

The study was approved by an appropriate institution (Ethical Committee of Gastroenterology of Hospital Senhora da Oliveira, Guimarães). All methods were performed in accordance with relevant guidelines and regulations, namely the ethical guidelines of the 1975 Declaration of Helsinki.

## Results

### Demographic, clinical, biochemical and endoscopic data

During the study period, at our center, a total of 56 CD patients were submitted to conventional IUS and CEUS with contrast SonoVue® directed to the terminal ileum, performed by a single operator. Six patients were excluded from the study: 1 with previous surgery evolving terminal ileum; 3 with inadequate ultrasonographic profile with reduced visualization and signal intensity; 2 with inadequate bowel preparation during ileocolonoscopy, in which terminal ileum evaluation was not possible. Fifty patients were included in the study. Included baseline demographic and clinical characteristics of patients are presented in Table 2. Most of the patients were female (n = 27; 54.0%), with mean age of 34 ± 12 years. Considering Montreal classification for CD [26], most of the patients had CD diagnosis established between 17 and 40 years (A2) (n = 40, 80.0%), isolated ileal disease (L1) (n = 30, 60.0%), and a nonstricturing, nonpenetrating behaviour (B1) (n = 22; 44.0%). Patients were in clinical remission (HBI ≥ 5) in 52.0% (n = 26) of cases.

**Table 2:** Baseline demographic and clinical characteristics of CD patients

Gender, female, n (%)	27 (54.0)
Age, mean ± SD, years	34 ± 12
Montreal classification [26], n (%)	
A1, < 16 years	2 (4.0)
A2, 17–40 years	40 (80.0)
A3, > 40 years	8 (16.0)
L1, ileal	30 (60.0)
L2, colonic	0
L3, ileocolonic	20 (40.0)
L4, upper gastrointestinal disease	0
B1, nonstricturing, nonpenetrating	22 (44.0)
B2, stricturing	17 (34.0)
B3, penetrating	11 (22.0)
HBI, mean ± SD	5 ± 4
Clinical remission (HBI < 5), n (%)	26 (52.0)
Clinically active disease (HBI ≥ 5), n (%)	24 (48.0)
SES-CD, mean ± SD	7 ± 4
Active disease (SES-CD ≥ 7), n (%)	30 (60.0)
Inactive disease (SES-CD < 7), n (%)	20 (40.0)
CRP, mean ± SD (mg/L)	21.2 ± 32.7
CRP > 3.0 mg/L, n (%)	42 (84.0)
Fecal calprotectin, mean ± SD (μg/g)	857 ± 610
Fecal calprotectin ≥ 150 μg/g, n (%)	38 (76.0)

*CD* Crohn’s disease; *SD* standard deviation; *HBI* Harvey Bradshaw Index; *SES-CD* Simple Endoscopic Score for CD; *CRP* C-reactive protein

Regarding biochemical data, patients had a mean CRP of 21.2 ± 32.7 mg/L and a mean fecal calprotectin of 857 ± 610 μg/g (Table 2).

Concerning endoscopic activity, patients had a mean segmental SES-CD applied to terminal ileum of 7 ± 4, most of the patients having active disease (n = 30; 60.0%) (Table 2).

### Ultrasonographic data

**Fig. 1:**
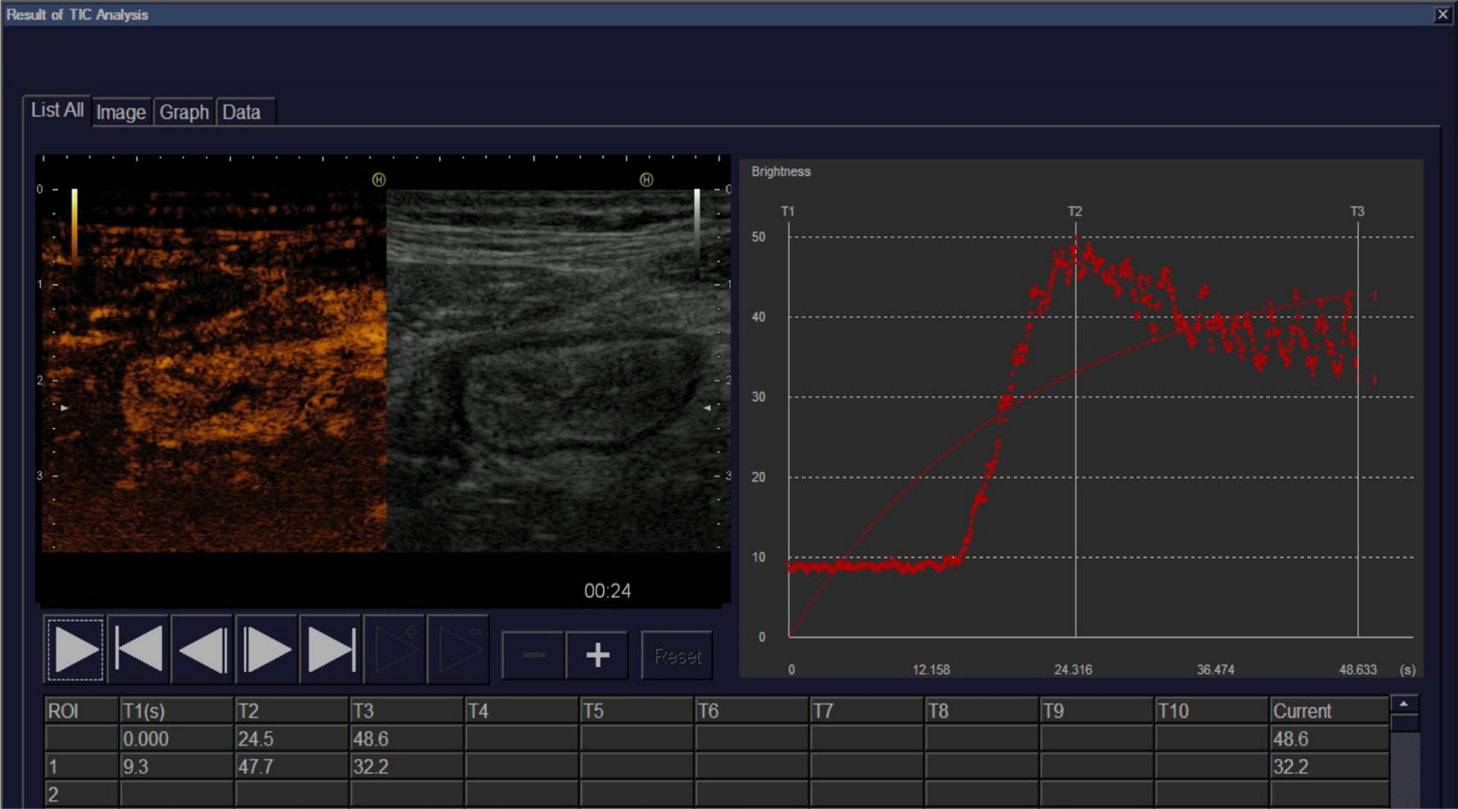
Contrast-enhanced ultrasound and peak intensity curve examination

An example of contrast-enhanced ultrasound and peak intensity curve examination of a patient is presented in Fig. 1. Ultrasonographic data are presented in Table 3. Patients had a mean BWT of 6.4 ± 1.9 mm, and the majority of patients had a BWT between 5.0–7.9 mm (n = 29; 58.0%). Most patients had preserved BWS (n = 34; 68.0%), and i-fat was present in most of them (n = 27; 54.0%). Regarding CDS, according to SUS-CD evaluation, most patients had perceptible 2–5 vessels/cm^2^ (n = 35; 70.0%), and according to IBUS-SAS evaluation, most patients had long doppler signals inside bowel (n = 22; 44.0%).

**Table 3:** Ultrasonographic findings

**BWT**, mean ± SD (mm)	6.4 ± 1.9
< 3.0 mm, n (%)	0
3.0–4.9 mm, n (%)	8 (16.0)
5.0–7.9 mm, n (%)	29 (58.0)
≥ 8.0 mm, n (%)	13 (26.0)
***CDS***	
**SUS-CD**	
No or single vessel, n (%)	1 (2.0)
2–5 vessels/cm^2^, n (%)	35 (70.0)
> 5 vessels/cm^2^, n (%)	14 (28.0)
**IBUS-SAS**	
Absent, n (%)	1 (2.0)
Short signals, n (%)	13 (26.0)
Long signals inside bowel, n (%)	22 (44.0)
Long signals inside and outside bowel, n (%)	14 (28.0)
**i-fat,** n (%)	
Absent	23 (46.0)
Present	27 (54.0)
**BWS**, n (%)	
Normal	34 (68.0)
Focal (≤ 3 cm)	10 (20.0)
Extensive (> 3 cm)	6 (12.0)
**SUS-CD**, mean ± SD	3.3 ± 1.0
**IBUS-SAS**, mean ± SD	56.2 ± 26.0
**Peak intensity (CEUS)**, mean ± SD	12.4 ± 12.1

*BWT* Bowel wall thickness; *CDS* Color Doppler signal; *i-fat* Inflammatory fat BWS Bowel wall stratification; *SUS-CD* Simple Ultrasound Activity Score for CD; *IBUS-SAS* International Bowel Ultrasound Segmental Activity Score; *CEUS* Contrast ultrasound

Patients had a mean SUS-CD score of 3.3 ± 1.0, a mean IBUS-SAS score of 56.2 ± 26.0, and a mean CEUS peak intensity of 12.4 ± 12.1.

No adverse effects with the contrast administration of CEUS were reported.

### Assessment of segmental SUS-CD and IBUS-SAS scores performance in correlating CD inflammatory activity

Segmental SUS-CD and IBUS-SAS scores performance in correlating CD inflammatory endoscopic activity is included in Table 5.

SUS-CD was not different between patients with active (SES-CD ≥ 7) or inactive endoscopic disease (SES-CD < 7) (3.5 ± 1.0 vs 3.1 ± 0.9, *p* = 0.15) (Table 4). SUS-CD had a poor accuracy to correlate endoscopic activity (AUC 0.62, 95% CI 0.45–0.78, *p* = 0.18) (Table 5). SUS-CD was not significantly different in patients with clinically active disease (HBI ≥ 5) versus clinically inactive disease (3.6 ± 1.1 vs 3.1 ± 0.82, *p* = 0.07), and was not statistically different between patients with fecal calprotectin ≥ 150 μg/g versus < 150 μg/g (3.5 ± 0.94 vs 2.8 ± 1.0, *p* = 0.06) and between patients with CRP > 3.0 mg/L versus ≤ 3.0 mg/L (3.5 ± 1.1 vs 2.9 ± 0.51, *p* = 0.07).

**Table 4:** Comparison of IUS scores and CEUS regarding CD inflammatory endoscopic activity

	Inactive disease (SES-CD < 7)	Active disease (SES-CD ≥ 7)	*p*
SUS-CD, mean ± SD	3.1 ± 0.9	3.5 ± 1.0	0.15
IBUS-SAS, mean ± SD	55.4 ± 23.2	59.9 ± 28.7	0.57
Peak intensity (CEUS), mean ± SD	7.2 ± 6.5	16.7 ± 14.1	**0.004**

P values in bold are statistically significant (*p* < 0.05)

*IUS* Intestinal ultrasound; *CEUS* Contrast ultrasound; *CD* Crohn’s disease; *SES-CD* Simple Endoscopic Score for CD; *SUS-CD* Simple Ultrasound Activity Score for CD; *IBUS-SAS* International Bowel Ultrasound Segmental Activity Score

**Table 5:** IUS scores and CEUS performance in correlating CD inflammatory endoscopic activity

	AUC (95% CI)	*p*
SUS-CD	0.62 (0.45–0.78)	0.18
IBUS-SAS	0.55 (0.38–0.72)	0.59
Peak intensity (CEUS)	0.80 (0.66–0.94)	**0.002**

P values in bold are statistically significant (*p* < 0.05)

*IUS* Intestinal ultrasound; *CEUS* Contrast ultrasound; *CD* Crohn’s disease; *AUC* area under the curve; *CI* confidence interval; *SUS-CD* Simple Ultrasound Activity Score for CD; *IBUS-SAS* International Bowel Ultrasound Segmental Activity Score

IBUS-SAS was not different between patients with active (SES-CD ≥ 7) or inactive disease (SES-CD < 7) (59.9 ± 28.7 vs 55.4 ± 23.2, *p* = 0.57) (Table 4). IBUS-SAS had a poor accuracy to correlate endoscopic activity (AUC 0.55, 95% CI 0.38–0.72, *p* = 0.59) (Table 5). IBUS-SAS was not significantly different in patients with clinically active disease (HBI ≥ 5) versus clinically inactive disease (66.2 ± 29.2 vs 52.3 ± 20.8, *p* = 0.06), and was not statistically different between patients with fecal calprotectin ≥ 150 μg/g versus < 150 μg/g (62.0 ± 25.4 vs 43.0 ± 24.0, *p* = 0.06) and between patients with CRP > 3.0 mg/L versus ≤ 3.0 mg/L (63.5 ± 26.3 vs 60.2 ± 30.2, *p* = 0.72).

### Assessment of CEUS performance in correlating CD inflammatory activity

**Fig. 2:**
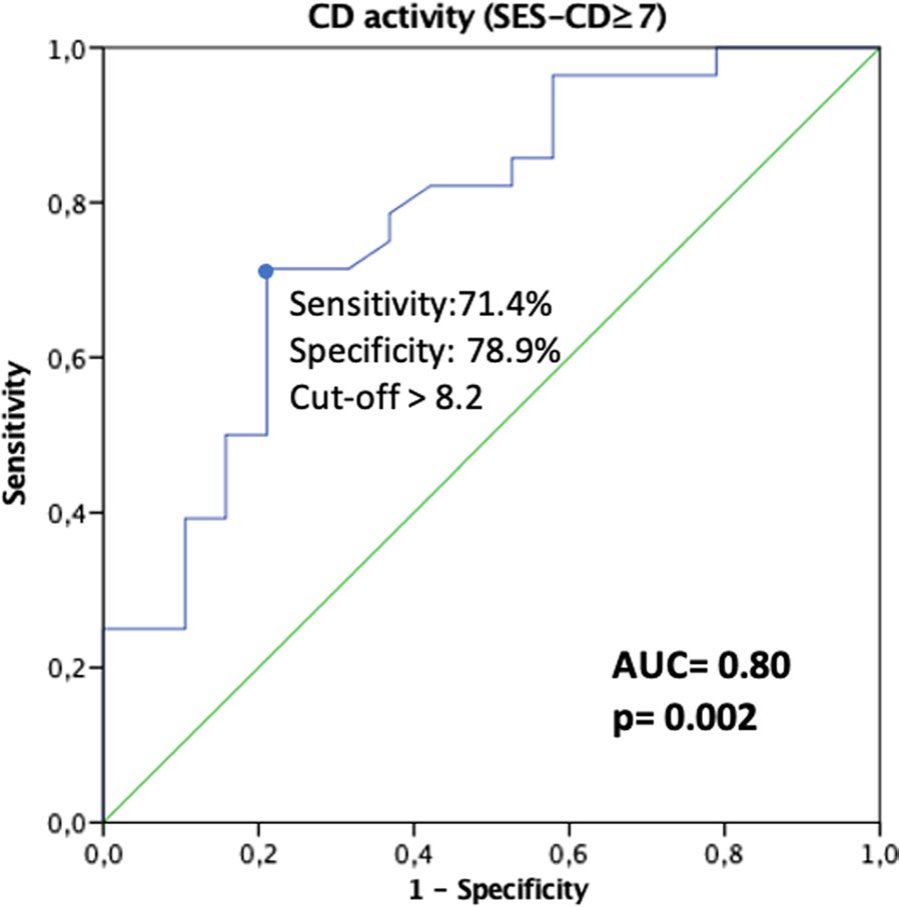
Peak intensity receiver operating characteristic curve with the optimal cut-off value for correlating CD inflammatory endoscopic activity

Peak intensity in CEUS was significantly different in patients with active or inactive endoscopic disease (16.7 ± 14.1 vs 7.2 ± 6.5, *p* = 0.004) (Table 4). CEUS performance in correlating CD inflammatory endoscopic activity is included in Table 5. Peak intensity in CEUS had a good accuracy to correlate endoscopic activity (AUC 0.80; 95% CI 0.66–0.94, *p* = 0.002). Our machine/investigator optimal cut-off of peak intensity to correlate active disease (SES-CD ≥ 7) was 8.2 with a sensitivity of 71.4% and a specificity of 78.9% (Fig. 2).

Peak intensity in CEUS was significantly different in patients with clinically active disease (HBI ≥ 5) versus clinically inactive disease (17 ± 8 vs 8 ± 7, *p* = 0.01).

Peak intensity in CEUS was not statistically different between patients with fecal calprotectin ≥ 150 μg/g versus < 150 μg/g (13.1 ± 12.7 vs 10.0 ± 9.2, *p* = 0.52) and between patients with CRP > 3.0 mg/L versus ≤ 3.0 mg/L (13.3 ± 13.5 vs 10.4 ± 6.3, *p* = 0.47).

## Discussion

Prompt detection of active CD is essential to early guide the therapeutic decisions and to avoid serious complications. However, due to the heterogeneous clinical presentation and disseminated nature of the disease, there is no gold standard for the diagnosis and evaluation of disease activity [25]. IUS is useful in follow-up examinations since it is non-invasive, easily accessible, inexpensive, and well-tolerated [15, 20]. Unlike computed tomography or MRE, IUS can be easily performed as a point of care scan by gastroenterologists to allow timely assessment of disease activity to guide clinical and therapeutic decisions. However, its usefulness in follow-up examinations in CD has not been fully established. IUS is operator dependent, and although several sonographic activity scores are available [36–42], there is a lack of validated reproducible scoring system, which limits its widespread clinical use. Recently, two new scores were published, the SUS-CD [21] and IBUS-SAS [22], in order to attempt to overcome the inaccuracies of the previous scores. The SUS-CD was validated and correlated well with moderate to severe endoscopic activity (SES-CD ≥ 7) at ileocolonoscopy (AUC 0.88), seems to be reproducible as it showed a low interobserver variability, and is applicable at different disease stages, since a heterogeneous CD population was analysed [21]. The IBUS-SAS [22] predicted CD inflammatory endoscopic activity and also demonstrated excellent reliability, although further external validation is required [22]. On the other hand, in our study, SUS-CD and IBUS-SAS were not able to accurately correlate endoscopic activity in CD assessed by SES-CD. However, this information is not directly comparable, since in our study only segmental SUS-CD and IBUS-SAS, with exclusive assessment of activity in terminal ileum have been included, differently from the cohorts in the publication of these scores, which included ileum, colon and rectum assessment. Future studies should address the accuracy of the IUS scores, according to inflammatory activity location.

In contrast to IUS scores, CEUS with peak intensity assessment showed a good diagnostic accuracy for active endoscopic inflammation. Recently, some studies suggest that transmural healing evaluated by cross-sectional imaging and ileocolonoscopy is superior to mucosa healing alone at ileocolonoscopy in predicting better long-term outcomes [43–45]. Since CEUS showed a good accuracy to correlate endoscopic activity, we speculate its potential to reduce the need for ileocolonoscopic examinations and to become a promissor tool in the era of transmural healing target. A systematic review and meta-analysis found a pooled sensitivity and specificity of CEUS in the detection of the acute phase of CD of 0.94 and 0.79, respectively, and found that B-mode IUS seems to be less sensitive than CEUS [25]. Guidelines by the European Federation of Societies for Ultrasound in Medicine and Biology indicate that adding CEUS to the routine diagnostic protocol improves reliability in estimating the activity of CD and that quantitative measurements of enhancement obtained by CEUS also correlate with activity [46]. However, no widely accepted consensus regarding enhancement parameters for the diagnosis of active CD have been proposed, and studies regarding CEUS were based on small numbers without any previous sample statistical calculations, compromising the evaluation of the diagnostic value of CEUS [46]. In our study, the optimal peak intensity cut-off in CEUS for correlating active disease was 8.2 with a sensitivity of 71.4% and a specificity of 78.9%. We highlight that this cut-off is a machine/operator dependent parameter, and so it may not be reproducible in other settings with distinct operator and ultrasound machine. In the same settings, the peak intensity can be used as a parameter in inflammatory activity monitoring/follow up during treatment. We emphasize that an advantage of CEUS is the generation of TICs as an objective parameter of bowel enhancement, as opposed to subjective IUS parameters included in the studied scores. Besides, although BWT is considered the most reliable sonographic feature that reflects inflammation, there are structural changes such as thickening of the ileon wall throughout the healing process that can persist, and some patients with quiescent disease continue to show bowel wall thickening in the absence of active inflammation. Thus, the IUS scores may erroneously classify fibrotic segments as inflamed lesions, putting patients at risk of receiving inadequate treatment [47]. Moreover, in some cases, CDS is not reliable due to patient body habitus or technical factors and can show a poor signal detection in a thickened with active inflammation bowel wall. In these situations, CEUS is a valuable tool often showing transmural enhancement and high CEUS parameters in cases of active disease where no color signal can be detected at all due to technical failure. On the other hand, in patients with long standing disease, where fat infiltration of the submucosal layer often creates thickened bowel wall with no CDS, chronic/quiescent disease needs to be differentiated from acute on chronic active disease [47]. These reasons may contribute to the fact that BWT and CDS, included in IUS scores, are not the best method to assess inflammatory activity, as opposed to CEUS.

Endoscopy remains the gold standard for assessing location, depth, and extent of inflammatory mucosal lesions in CD and several endoscopic scoring systems have been developed, such as SES-CD [21] and Crohn’s Disease Endoscopic Index of Severity (CDEIS) [48]. However, although validated, they are complex and difficult to apply in clinical practice [49]. Therefore, it is essential the emergence of simple and non-invasive methods of monitoring the CD activity, especially in the frequently young patient with IBD who needs several follow-up examinations throughout the chronic course of their disease. Thus, IUS plays a promising role for this purpose, being a well-tolerated examination, with an easy repeatability, absence of sedation or preparation. We highlight that, since there is no validated optimal SES-CD cut-off score and the quantification of disease severity has likewise not been standardised yet [50], this can be a limitation of our study regarding the definition of active disease at ileocolonoscopy.

Our study showed that SUS-CD and IBUS-SAS were not able to correlate clinically active disease (HBI ≥ 5). It is known that clinical indices such as the CD Activity Index (CDAI) and HBI correlate poorly with mucosal inflammation [5, 6], which may partially explain these results. However, CEUS can be a more reliable indicator of clinical active disease, since peak intensity in CEUS had a good accuracy in correlating clinically active disease. According to the concept of “treat-to-target” strategy, deep remission (defined by both clinical and endoscopic remission) has become a new therapeutic goal, significantly improving patients’ long-term outcome [51]. As CEUS correlates with clinical and endoscopic activity maybe it could also be a promissor parameter representative of this endpoint.

SUS-CD and IBUS-SAS had a fair accuracy, and peak intensity in CEUS had a poor accuracy to correlate elevated fecal calprotectin and elevated CRP. Although inflammation biomarkers are useful in diagnosis and monitoring of CD activity, they have limited accuracy and specificity, and cannot evaluate disease location and extension, which can explain these results [13]. Besides, some studies suggested that fecal calprotectin may be less sensitive in isolated small bowel disease [52–54], and we only analysed terminal ileum activity. Even though, it is conceivable that combinations of inflammation biomarkers and IUS scores or CEUS might enhance sensitivity for DC activity assessment, and future studies could address this issue.

We emphasize that our study has some limitations, such as its retrospective nature and small sample. The small sample is explained by the fact that only CD patients that performed biochemical and endoscopic examinations with a maximum interval of 1 month until the ultrasonographic examination, and with no changes in medical therapy during this period were included. In clinical practice this does not occur regularly, but we only considered these patients in our analysis to limit potential bias. However, we highlight that there was only one patient that performed the ultrasonographic and ileocolonoscopic examinations with an interval of 1 month, the remaining had a shorter interval between examinations. Moreover, we did not analyze histological activity, that would be relevant to understand its correlation with ultrasound activity. Besides, due to the retrospective character of our study we cannot use the concept “blinded” regarding the ultrasound operator to the results of the ileocolonoscopy. However, we guarantee that the ultrasound operator was not the same that performed ileocolonoscopy examinations and the ileocolonocopy examinations were performed after ultrasonographic examinations by an operator without experience in ultrasound. Despite these limitations, we believe that our study is relevant because it can contribute in clinical practice to the improvement of CD activity monitoring.

In conclusion, in an era where the paradigm of mucosal healing is changing to transmural healing, where there is a lack of agreement of patient’s symptoms with disease activity, where endoscopic activity scores are complex and difficult to apply and with the emergence of several therapies leading to frequent imaging surveillance, CEUS is a promising non-invasive emerging method, that showed a good accuracy to correlate clinical and endoscopic activity, categorically superior to IUS scores. Therefore, CEUS should be increasingly integrated in the ultrasonographic evaluation in CD, potentially reducing the need for endoscopic examinations. However, since evidence for the routine use of CEUS is based on small study groups and with significant methodological heterogeneity [25], there is still a need for large prospective studies on the role of CEUS in active CD detection that would help in introducing the method into everyday practice.

## Data Availability

The datasets used and/or analysed during the current study are available from the corresponding author on reasonable request according to institutional policies.

## Abbreviations

IUS: Intestinal ultrasound
CD: Crohn’s disease
CT: Computed tomography
MRI: Magnetic resonance imaging
MRE: Magnetic resonance enterography
SUS-CD: Simple Ultrasound Activity Score for Crohn’s disease
IBUS-SAS: International Bowel Ultrasound Segmental Activity Score
CEUS: Contrast-enhanced ultrasound
SES-CD: Simple Endoscopic Score for Crohn’s disease
CRP: C-reactive protein
BWT: Bowel wall thickness
CDS: Colour Doppler signal
i-fat: Inflammatory mesenteric fat
HBI: Harvey Bradshaw Index
TIC: Time-intensity curve
SD: Standard deviation
AUCs: Area under the receiver operating characteristic curves
Cis: Confidence intervals
CDEIS: Crohn’s Disease Endoscopic Index of Severity
CDAI: Crohn’s Disease Activity Index
